# Contrasting Ecosystem-Effects of Morphologically Similar Copepods

**DOI:** 10.1371/journal.pone.0026700

**Published:** 2011-11-29

**Authors:** Blake Matthews, Stephen Hausch, Christian Winter, Curtis A. Suttle, Jonathan B. Shurin

**Affiliations:** 1 Center for Ecology, Evolution and Biogeochemistry, Aquatic Ecology Department, Eawag: Swiss Federal Institute of Aquatic Science and Technology, Kastanienbaum, Luzern, Switzerland; 2 Department of Biological Sciences, University of Calgary, Calgary, Alberta, Canada; 3 Department of Marine Biology, University of Vienna, Vienna, Austria; 4 Departments of Earth and Ocean Sciences, Microbiology and Immunology, and Botany, University of British Columbia, British Columbia, Canada; 5 Section of Ecology, Behavior and Evolution, University of California San Diego, La Jolla, California, United States of America; University of British Columbia, Canada

## Abstract

Organisms alter the biotic and abiotic conditions of ecosystems. They can modulate the availability of resources to other species (ecosystem engineering) and shape selection pressures on other organisms (niche construction). Very little is known about how the engineering effects of organisms vary among and within species, and, as a result, the ecosystem consequences of species diversification and phenotypic evolution are poorly understood. Here, using a common gardening experiment, we test whether morphologically similar species and populations of Diaptomidae copepods (*Leptodiaptomus ashlandi, Hesperodiaptomus franciscanus, Skistodiaptomus oregonensis*) have similar or different effects on the structure and function of freshwater ecosystems. We found that copepod species had contrasting effects on algal biomass, ammonium concentrations, and sedimentation rates, and that copepod populations had contrasting effects on prokaryote abundance, sedimentation rates, and gross primary productivity. The average size of ecosystem-effect contrasts between species was similar to those between populations, and was comparable to those between fish species and populations measured in previous common gardening experiments. Our results suggest that subtle morphological variation among and within species can cause multifarious and divergent ecosystem-effects. We conclude that using morphological trait variation to assess the functional similarity of organisms may underestimate the importance of species and population diversity for ecosystem functioning.

## Introduction

Organisms can broadly affect the physical, chemical, and biological properties of ecosystems, and can influence the fluxes of matter and energy through ecosystems (i.e. ecosystem functions) [1]–[3]. The ecosystem-effects of organisms are mediated by both trophic and non-trophic interactions [4], [5]. Primary producers, for example, can provision habitat structure and moderate abiotic stress [6], [7], whereas consumers can affect the flux of biomass along food chains and the rate of nutrient recycling in the environment [2], [8], [9]. The ecosystem-effects of organisms can arise via ecosystem engineering, whereby organisms alter the availability of resources to other organisms [2], [10]. Ecosystem engineering is an important mechanism of niche construction, the process by which organisms modify their environment and alter selective regimes of future generations [11], [12]. Although the ecosystem- and engineering-effects of species are potentially large, the underlying ecological and evolutionary causes of variation in their magnitude are poorly understood [13], [14].

A useful starting point for predicting the ecosystem-effects of different organisms is to consider what evolutionary processes have caused the phenotypic variation within and among species. For example, are the phenotypic traits that underlie species' ecosystem-effects also a target of natural selection? In adaptive radiations, for example, the traits under divergent selection between species are often those used to exploit resources in the natural environment [15]. In the adaptive radiation of threespine stickleback *Gasterosteus aculeatus*, for example, we would predict that divergence in foraging morphology and feeding behavior between species might cause strong and contrasting effects on ecosystems [13]. Whereas in non-adaptive radiations reproductive isolation can build up between species either independently from or in the absence of divergence in ecological and life-history traits. In the radiation of damselflies in North America, for example, species differ primarily in the morphological variation of male reproductive structures [16]. In such cases, the phenotypic divergence in mating traits, possibly resulting from sexual selection [17], will unlikely cause organisms to have contrasting ecosystem-effects. In general, the relative importance of natural and sexual selection in driving phenotypic evolution in species radiations will influence variability in the ecosystem consequences of phenotypic variation; however, experimental tests of these ideas are rare.

Common gardening experiments [18] are an increasingly popular way to investigate whether organisms with different phenotypes have contrasting effects on ecosystems [13], [19], [20]. In a common gardening experiment the phenotypes of organisms are held constant for each ecosystem type, in order to quantify how much variation in ecosystem properties and functions is attributable to phenotypic differences among organisms [18]. For example, Harmon and colleagues found that stickleback populations with different phenotypes had contrasting effects on zooplankton community structure, gross primary productivity, and rates of light extinction [13]. Such experiments are particularly useful for studying the ecosystem consequences of organisms with different evolutionary histories [13], [19], [20].

Previous common gardening experiments have found that organisms with different phenotypes can cause a variety of different ecosystem-effects [13], [19], [20], but is the magnitude of phenotypic divergence between species or populations a good predictor of the resulting size of ecosystem-effect contrasts? To date, previous studies have used organisms as experimental treatments that clearly differ in several functional traits that could plausibly cause different effects on ecosystems, such as foraging morphology, life-history, and behavior. For example, different ecotypes of alewives (*Alosa pseudoharengus*), which vary in their gill raker morphology, have contrasting effects on the species composition and size structure of zooplankton communities in lakes [19]. Similarly, guppy populations (*Poecilia reticulata*) with different life-histories and feeding behaviors have contrasting effects on rates of primary productivity in streams [20]. However, to test the generality of the relationship between phenotypic divergence and the resulting divergence in ecosystem-effects we need comparable common gardening experiments that use groups of organisms that span a broad gradient of phenotypic differentiation. One can do this by using species from both adaptive and non-adaptive radiations as experimental treatments. In the current study, we set out to (i) do a common gardening experiment to measure the ecosystem-effect contrasts for a group of morphologically similar species and populations, and (ii) compare the size of these contrasts with other common gardening experiments that used organisms with more divergent phenotypes [13], [19].

**Figure 1:**
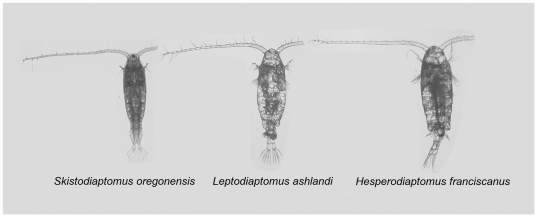
Adult females from three species of Diaptomidae copepods.

**Figure 2:**
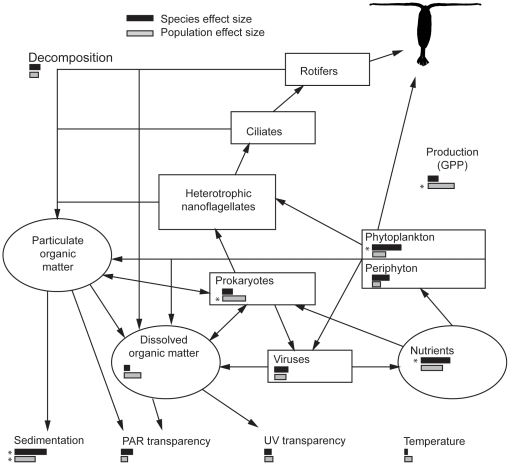
A depiction of the potential ecosystem-effects of copepods. Copepods might influence ecosystem functions as well as the biological, chemical, and physical properties of ecosystems. The bars are proportional to the average standardized effect sizes (Cohen's 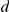) for species contrasts (black bars) and population contrasts (grey bars), as summarized in Table 2. Stars indicate if the ecosystem metric had at least one significant contrast (at the level of 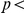
0.05), as indicated in Table 2.

We chose a radiation of freshwater Diaptomidae copepods for our common gardening experiment because they exhibit little morphological divergence among populations and species [21]–[24]. We chose three copepod species (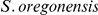, 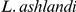, and 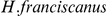) that represent three different genera in the Diaptomidae family (Figure 1) and that have a similar range of body size (0.9–1.5 mm: [25]). Unfortunately, there is very little quantitative information about the diet and functional trait differentiation among and within freshwater copepod species [25]. 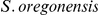 and 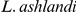 have very similar life histories and feeding preferences [25], but it is unknown whether they have similar impacts on ecosystems (Figure 2). Virtually nothing is known about the biology of 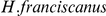 except that it is morphologically very similar to 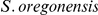 [26]. For this reason, copepod taxonomists originally grouped 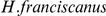 in the same genus as 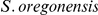 [27], but later realized that characters of the distal pad of the left exopods of males were an important feature distinguishing these two groups [28]. Genetic analyses have subsequently confirmed that 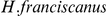 is not a member of the *Skistodiaptomus* genus [26]. This history of taxonomic confusion in the Diaptomidae family attests to the morphological similarity of these copepod species. If such morphological similarity among Diaptomidae species also implies functional equivalency in ecosystems, then we would predict that different species and populations would have similar ecosystem-effects. Alternatively, cryptic or unknown divergence in their diet, species interactions, and nutrient excretion, resulting from local adaptation, for example, could drive variation in their effects on other aquatic organisms or on the physical and chemical environment.

## Materials and Methods

No specific permits were required for the described field studies. All collections were taken from public property and did not include any endangered or protected species.

### Description of the study system

Copepods often dominate the metazoan biomass of open-water marine and freshwater environments [29], [30] and can have a variety of effects on the properties and functions of aquatic ecosystems (Figure 2). Diaptomidae copepods are omnivorous, and feed readily on both the algal and microbial food chains of lake food webs [31]. Despite their prominence in aquatic food webs little is known about how species and populations differ in their dietary preferences [25], [32], [33], and whether they are functionally equivalent in aquatic ecosystems.

The radiation of Diaptomidae copepods is a useful model system to investigate the ecosystem consequences of species that appear morphologically similar (Figure 1), and for which there is evidence of morphological stasis among genetically divergent groups of species [24]. As in the radiation of damselflies in North America [16], copepods species show prominent morphological differences in mating traits (e.g. male reproductive structures) [34], suggesting that sexual selection has been an important process driving species diversification [17], [24].

We sampled copepods from inland lakes in British Columbia, Canada, within a geographical area of about three hundred square kilometres. 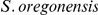, 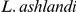, and 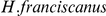 rarely co-occur in the same lake, often constitute a large proportion of zooplankton biomass, and each occur in lakes over a broad range of environmental gradients [35]. In general, copepods show strong spatial structure in their species distribution across the landscape that is not accounted for by variation in the abiotic and biotic environment of lakes [36]. This suggests that variation in the dispersal and colonization abilities of different Diaptomidae copepods is the more likely cause of their spatial structure, rather than species-specific differences to their abilities to exploit resources in different lake environments [35]. This hypothesis, however, is difficult to confirm because there is very limited quantitative information about how Diaptomidae species differ in their feeding preferences, their life histories, and their relative fitness under different environmental conditions [25].

### Experimental design of the common gardening experiment

We measured the ecosystem-effects of five populations of three different Diaptomidae species (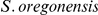, 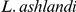, and 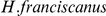). We filled forty-eight outdoor tanks (Diameter = 0.7 m, Height = 0.9 m, Volume 320 L) with municipal drinking water originating from several nearby oligotrophic lakes. We added leaf leachate (from fresh alder leaves) and nutrients (KH
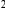
PO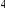 and NaNO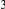) to reach a final concentration of dissolved organic carbon of 3.1 mg C/L (SD = 0.7, N = 48), and 15 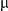
g/L of P and 240 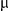
g/L of N. This level of nutrient loading for the environment matched the level of productivity that all three species are known to experience within their respective geographic ranges [35]. Tanks were left for three weeks prior to their inoculation with copepods. Because these tanks were only an abstraction of the complexity of natural systems, we did not aim to match the specific observed ecosystem responses to lake environments. We acknowledge that information on the selective environment of these copepods in the natural environment would be useful for disentangling the specific mechanisms that cause contrasting ecosystem-effects of different species, but this was beyond the scope of the current paper.

**Figure 3:**
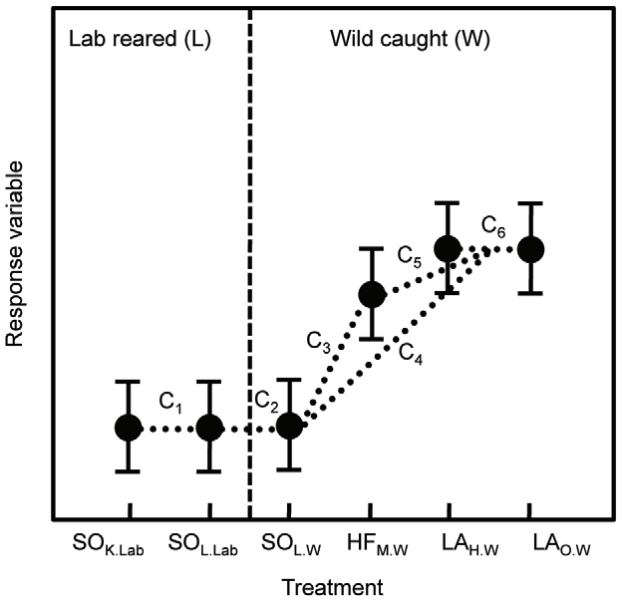
A schematic of the contrasts used in the copepod experiment. See the text for an explanation of the contrasts. The treatment labels indicate the species (uppercase), source population (subscript before the period), and rearing environment (subscript after the period). The three species are: 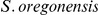 (SO), 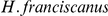 (HF), and 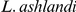 (LA). The origins of the source populations are: Killarney Lake (K), Loon Lake (L), Mitchell Lake (M), Harrison Lake (H), and Osoyoos Lake (O). The rearing environments are: Lab-reared (Lab), and Wild-caught (W).

We established eight replicates for each of the following six copepod treatments (Figure 3): two treatments consisting of lab-reared populations of 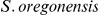 (SO) from either Killarney Lake (SO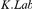, 49

23′30″N, 123

21′18″W) or Loon Lake (SO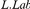), and four treatments consisting of wild-caught (W) populations of either 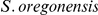 from Loon Lake (SO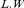, 49

18′18″N, 122

35′21″W), 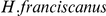 from Mitchell Lake (HF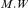, 48

30′43″N, 123

30′17″W), and 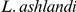 from Harrison Lake (LA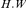, 49

20′31″N, 121

45′05″W) or Osoyoos Lake (LA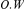, 49

02′00″N, 119

27′33″W). We initially attempted to rear all three species in the lab, to achieve a more balanced experimental design, but only 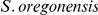 grew in sufficient densities given the timing of our experiment. To establish the lab-reared population treatments we collected 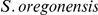 from Killarney and Loon Lake on July 16–18, 2007, narcotized hundreds of females, removed their egg clutches, and transferred three clutches to each of forty-eight one liter glass jars filled with COMBO medium [37]. We reared copepods to adults on *Cryptomonas erosa*, and when they started to produce egg clutches we began our sampling campaign to establish our ‘wild-caught’ copepod treatments (Figure 3). From Aug 8–11th, 2007, we collected 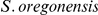 from Loon Lake, 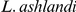 from Harrison Lake and Osoyoos Lake, and 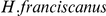 from Mitchell Lake. Because of the strong spatial structure of these copepod species we were unable to collect populations that were geographically close to one another. The experiment began on Aug 12th, 2007, when each tank received forty adult copepods (20 males and 20 females), either from the lab-reared cultures or from the wild-caught populations. Although we controlled for sex ratio, we could not control for variation in clutch size of the females, many of which were carrying egg clutches.

To analyze the ecosystem differences among copepod treatments we made the following six contrasts, where each contrast is the difference between the indicated treatments (see Figure 3). LA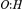 includes all the tanks from both LA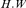 and LA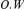 treatments.

1. SO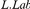-SO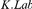 : between lab populations (*Lab*) of 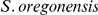 (from lakes 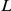 and 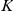)
2. SO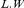-SO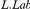 : between wild (*W*) and lab populations of 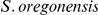 (from 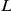)
3. HF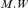-SO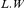 : between species of 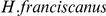 (from 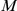) and 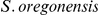 (from 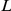)
4. LA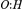-SO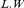 : between species of 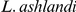 (from *H* and *O*) and 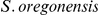 (from 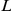)
5. LA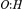-HF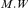 : between species of 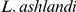 (from *H* and *O*) and 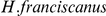 (from 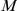)
6. LA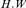-LA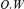 : between wild populations of 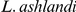 (from *H* and *O*)

Contrasts three to five are species comparisons (C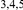), contrast one and six are population comparisons (C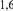), and contrast two is a rearing environment comparison (C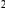).

### Ecosystem response variables

We measured several ecosystem metrics (EMs), either weekly for six weeks or once at the end of the experiment (Table 1). We intentionally chose parameters that vary widely in their likelihood to be influenced by copepods, so as to achieve a broad multivariate description of potential ecosystem-effects (Figure 2).

**Table 1:** Statistical analysis of ecosystem metrics.

Ecosystem metric	F 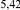	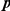	R 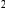	Median	IQR	Flatness	Levels	Paralellism	LDA 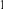	LDA 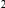	LDA 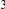
*Biological properties*											
Algal biomass ( 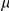 g Chl- 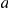 L 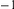 )	5.88	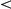 0.001	0.34	1.4	1.5	0.003	0.61	0.52	−0.61	1.02	0.19
Prokaryote abundance (10 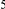 L 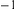 )	2.80	0.03	0.16	11.9	5.0	0.08	0.71	0.26	−0.20	0.58	−0.47
Virus abundance (10 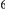 L 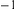 )	0.78	0.57	0.01	27.7	17.5	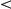 0.001	0.21	0.78	0.36	−0.50	−0.91
Periphyton ( 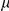 g Chl- 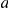 cm 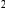 )	0.86	0.51	0.02	3.9	8.3	–	–	–	0.11	−0.13	0.7
*Chemical and physical properties*											
Temperature (  C)	0.14	0.98	0.02	16.9	0.6	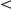 0.001	0.92	0.31	0.26	−0.67	−0.59
Ammonia ( 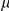 g L 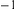 )	2.97	0.02	0.26	2.8	27.3	0.004	0.21	0.24	0.2	0.78	0.73
DOC (mg C 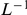 )	1.71	0.15	0.17	2.6	0.7	–	–	–	0.05	0.08	−0.5
PAR extinction ( 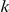 )	0.51	0.77	0.06	−1.3	0.1	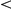 0.001	0.52	0.50	−0.13	0.31	0.51
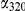	0.64	0.66	0.07	2.4	0.4	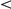 0.001	0.53	0.61	0.004	−0.19	−0.29
*Ecosystem functions*											
GPP (mg O 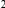 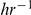 )	3.01	0.02	0.26	1.3	0.3	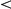 0.001	0.001	0.26	0.36	0.58	0.45
Sedimentation (mg 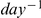 )	3.74	0.007	0.33	0.6	0.2	–	–	–	0.77	0.18	−0.31
Decomposition ( 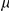 g 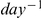 )	0.84	0.52	0.09	2.4	1.0	–	–	–	0.46	−0.33	−0.81

Results from the ANOVA at the end of the experiment, using log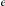 transformed data. The median and interquartile range (IQR) are reported for the last sampling date in the original units of the metric (i.e. not log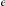 transformed). The ‘Flatness ’, ‘Levels ’, and ‘Parallelism’ columns show the p-values for each of these tests in the profile analysis (see text). Metrics without these tests were only measured once at the end of the experiment. PAR is photosynthetically active radiation, 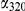 is the absorption co-efficient at 320 nm. Loadings for the first three axes from the linear discriminant analysis (LDA) explain 20%, 19%, and 15% of the discriminant function, respectively.

#### Biological properties

Algal biomass was estimated by filtering water through GF/F filters (Whatman) with a nominal pore size of 0.8 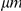, extracting the filters with 95% ethanol at 4

C overnight, and analyzing the concentration of chlorophyll-*a* (Chl-*a*) on a Trilogy fluorometer (Turner Designs) with the non-acidified module. Prokaryote and virus abundances were enumerated with a FACS-Calibur flow cytometer (Beckton Dickinson). Water samples (2 ml) were fixed with glutaraldehyde (0.5% final concentration), shock frozen in liquid nitrogen and stored at −80

C. Cells and virus particles were stained with SYBR green I prior to flow cytometry (FCM) [38]. Periphyton biomass was measured using 16 cm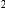 ceramic tiles placed in the bottom of the tanks at the beginning of the experiment. Periphyton was scrubbed from the tiles with a wire brush and rinsed with distilled water, and the solution was filtered onto GF/F filters (Whatman) and analyzed for Chl-*a* as with the phytoplankton samples.

#### Physical and chemical properties

Ammonium concentrations (NH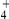) were analyzed on a Trilogy fluorometer (Turner Designs) following [39]. Samples for dissolved organic carbon analysis were filtered through ashed GF/F filters (Whatman) and analyzed on a Shimadzu 5000 TOC analyzer. Using the same samples, we measured absorption coefficients in 1-cm path-length quartz cells using a Cary 50 (Varian) UV-scanning spectrophotometer. Samples were scanned at 1 nm increments, and absorption coefficients were calculated as: 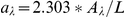, where 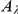 is the optical density for wavelength 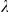 and 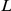 is the cell path length in meters. We chose the absorption coefficient at 320 nm (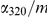) to compare the light environment among tanks because it is at the boundary of UV-B (280–320) and UV-A (320–400) and is a standard method to characterize the light environment of lakes. We measured attenuation of photosynthetically available radiation (PAR: 400–700 nm, 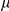
mols/s m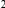) using a 4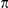 quantum sensor (LI-COR LI-193). A light extinction coefficient (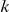) was calculated for each tank as the slope of the relationship between depth (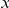) and ln(PAR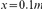/PAR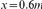), such that high 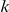 values are associated with low light penetration through the water column.

#### Ecosystem functions

Gross primary productivity (GPP) was estimated using diurnal changes in oxygen levels [40]. Dissolved oxygen (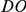) measurements were taken with an oxygen probe (YSI, Model 58) at sunrise (*t* 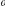), sunset (*t* 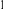), and the following sunrise (*t* 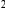), and GPP was calculated every week as (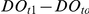)+(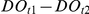). Sedimentation rate was calculated as the amount of dry mass per day that accumulated in glass jars placed at the bottom of each tank. Decomposition rate was measured as the weight loss of dry alder leaves.

### Statistical analysis

We used profile analysis (PA) to evaluate whether the time course of ecosystem changes differed among copepod species and populations. PA is an alternative to repeated-measures ANOVA (RM-ANOVA) that is well-suited to time series data [41]. PA involves three tests that are analogous to the standard tests from RM-ANOVA [41]: flatness, which tests the null hypothesis that all profiles show no change through time (similar to a “Time” effect in RM-ANOVA); levels, which tests whether profiles differ in their average values among treatments (similar to a “treatment” effect in RM-ANOVA); and parallelism, which tests whether profiles are parallel to each other (similar to a “time*treatment” effect). An advantage of profile analysis (over RM-ANOVA) is that it does not require the assumption of sphericity of the variance-covariance matrix. Violations of sphericity in RM-ANOVA designs are common, and in such cases profile analysis has greater power than tests that are adjusted for sphericity violations [41]. A drawback of PA is that it has low power when there are few repeated sampling events, and so for this reason we used ANVOA to analyze the results from the last sampling date.

In our common gardening experiment, significant flatness tests have a rather trivial explanation because they indicate that ecosystem metrics change over time in response to external forcing by changes in temperature, rainfall, and incident radiation. Significant levels and parallelism tests are more interesting, because they indicate that copepods differentially modify their environment by affecting the average values and trajectories of different ecosystem metrics, independent of externally driven environmental forcing. Such evidence suggests that organismal diversity can affect the divergence of identical ecosystem through time. It does not necessarily imply that variation in species composition will explain the observed environmental differences among lakes distributed across a environmentally heterogeneous landscape.

We used a multivariate analysis to compare the overall ecosystem-effects of different copepod treatments at the end of the experiment on a common scale. We calculated standardized z-scores for each of the EM across all 48 tanks, and used this matrix as the input for a Linear Discriminant Analysis (LDA). From the LDA we extracted five canonical axes of variation, which we used to calculate the average euclidean distance (ED) between the groupings of tanks specified by our six contrasts of interest. To determine the statistical significance of the observed EDs for each contrast, we generated a test distribution of EDs by randomizing the association of tanks with their treatments and repeating the above procedure 1000 times. This approach allowed us to evaluate the relative size and significance of each contrast using the entire matrix of ecosystem metrics. A drawback of this approach is that all ecosystem metrics are given an equal weight, regardless of their likelihood to be influenced by copepods (see Figure 2). For this reason, we also did a univariate analysis of each ecosystem metric.

We did an ANOVA of each EM, using log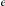 transformed data, measured on the last sampling date. We used a Bartlett test to confirm homogeneity of variance for the residuals of the ANOVA model, and used Q-Q plots to evaluate deviations from normality. We also calculated standardized effect sizes (Cohen's *d*; [42]) for each contrast and EM (Table 2), and used a paired t-test (N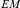 = 12) to examine whether the average size of contrasts between species was different than those between populations. We used Cohen's 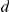 to compare our results to previous studies [13], [43].

**Table 2:** Analysis of contrasts from the copepod common gardening experiment.

Contrast	Algae 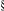	Prok	Virus	Peri 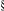	Temp 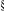	Ammonia 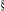	DOC 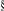	PAR 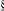	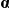 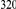 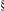	GPP 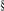	Sed	Decomp 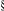	Uni-avg	Multi-avg
C 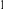 (pop)	0.42	0.66	−0.85	−0.39	0.14	−**0.93**	−0.29	−0.35	−0.28	−**1.33** **	−**1.05** *	−0.20	0.57	1.29
C 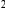 (env)	−0.24	−0.56	0.13	−0.73	0.03	−**0.84** *	−**1.02**	−0.36	0.03	−**1.77** **	−**0.97** *	−0.16	0.56	2.11**
C 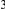 (spp)	−**1.34** *	−0.34	−0.41	**0.98**	0.16	**1.38** **	−0.31	−0.33	0.10	0.54	0.74	0.35	0.56	0.80
C 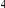 (spp)	0.27	0.31	−**0.77**	0.61	0.04	0.06	−0.02	0.35	0.37	0.11	−0.88	−0.22	0.35	1.08
C 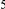 (spp)	**1.54** **	0.51	−0.36	−0.33	−0.14	−**1.81** **	0.26	0.60	0.29	−0.48	−**1.85** **	−0.61	0.74	2.62**
C 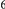 (pop)	−0.58	**1.09** *	−0.02	−0.24	−0.43	0.71	0.98	0.15	0.34	−0.66	0.51	0.46	0.51	0.14
All avg	0.73	0.58	0.42	0.55	0.16	0.95	0.48	0.36	0.23	0.82	1.0	0.33	0.55	
Spp avg	1.1	0.38	0.51	0.64	0.11	1.1	0.20	0.43	0.25	0.38	1.2	0.39	0.55	
Pop avg	0.50	0.88	0.43	0.31	0.28	0.82	0.64	0.25	0.31	1.0	0.78	0.33	0.54	

The standardized effect sizes (Cohen's *d*) for each ecosystem metric are calculated for each contrast illustrated in Figure 3, and are listed by columns in the same order as they appear in the rows of Table 1. The averages (avg) for each contrast and ecosystem metric are based on the absolute values of effect sizes. Contrasts were classified as either between species (spp), populations (pop), or rearing environments (env). The last two columns define the univariate average (Uni-avg), and the multivariate average (Multi-avg: from the LDA analysis). Values in bold indicate that the contrast was significant, which was assessed by post hoc analysis for each ecosystem metric and with a randomization for the multivariate average (see text). P-values are indicated as follows: , 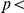
0.1;

*, 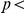
0.05;

**
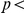
0.01.

The 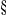 symbol indicates parameters used to compare with a previous stickleback experiment (see Table 3).

## Results

All the ecosystem metrics (EMs), with the exception of prokaryote abundance, changed significantly over time (Flatness test, Table 1). The level of Gross Primary Productivity (GPP) was significantly different among copepod treatments (Levels test, Table 1), but there were no significant effects of treatment on any of the time courses of the EMs (Parallelism test, Table 1). By the end of the experiment, however, treatments differed with respect to a multivariate characterization of the ecosystems (MANOVA: Wilk's 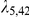 = 0.16, 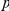 = 0.001; Mauchly's sphericity assumption: W = 0.34, 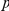 = 0.19). In a Linear Discriminant Analysis (LDA), the first three axes were significantly different among treatments (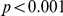) and explained 54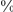 of the variance accounted for by the discriminant function (Table 1). Using all five LDA axes, we found that the euclidean distance (ED) was largest between the treatments with 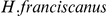 and 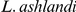 (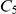, Table 2). Surprisingly, the same population of 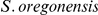 from Loon Lake reared under different environments (i.e. lab versus wild) had contrasting ecosystem-effects (Table 2). The rank of the contrast sizes (in terms of their ED) was as follows, 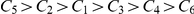, where C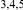 are species contrasts, C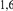 are population contrasts, and C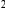 is a rearing environment contrast (Table 2).

**Figure 4:**
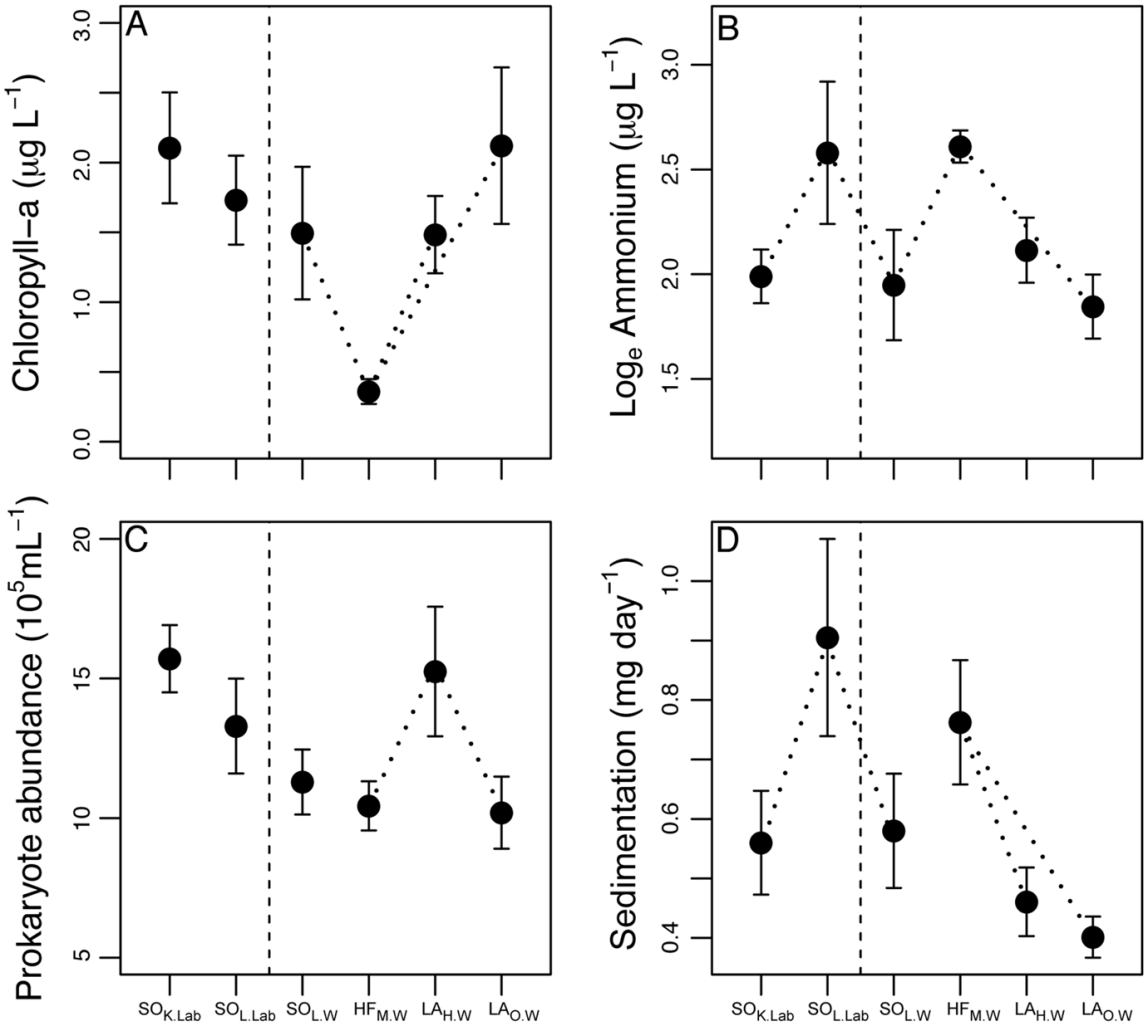
A summary of the ecosystem properties and functions that were influence by copepods. The vertical dashed line separates copepods that were either raised in the lab (left of line) or wild-caught in the field (right of line). The dotted lines connect treatments that are significantly different, based on Tukey's Post hoc contrasts (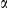 = 0.05). See Table 2 to see which specific contrasts (defined in Figure 3) were significant.

To investigate ecosystem divergence among copepod treatments in more detail, we examined our six contrasts separately for each ecosystem metric (Table 2). By the end of the experiment, the ecosystems associated with different treatments differed in their biological, chemical and physical properties, as well as in their functions (Figure 4, Table 2). Overall, the number of significant differences among the planned contrasts (11 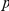-values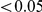) was higher than expected by chance (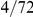 expected with 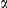 = 0.05; Table 2).

The EMs that led to the largest contrasts between treatments were ammonium concentrations, GPP, sedimentation rate, and algal biomass (Figure 4B), whereas EMs such as temperature, UV absorption, and decomposition rates had low average effect sizes across all contrasts (Table 2). Some EMs differed strongly between species while others differed between populations (Figure 2, Figure 4B). Averaging across each ecosystem metric individually (N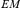 = 12), the size of ecosystem contrasts between species (mean = 0.55, range = 0.16–1.0) and between populations (mean = 0.54, range = 0.11–1.2) was remarkably similar (paired t-test: t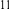 = −0.04, 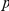 = 0.97; Figure 2; Table 2: last three rows). Overall, the univariate analysis (Table 2) led to a similar ranking of contrast sizes as from the multivariate analysis (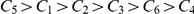).

## Discussion

Modifying the environment is an important consequence of an organism's livelihood that can have implications for the evolutionary and ecological dynamics of ecosystems [2], [12]. Here, we examined the relative size and nature of ecosystem-effects of three copepod species from a radiation with limited morphological differentiation among species [24], [26]. We found morphologically similar species and populations of copepods differed in their overall influence (in a multivariate sense) on aquatic ecosystems, and in their specific effects on the biological properties, chemical and physical properties, and functions of aquatic ecosystems (Figure 4, Table 2). The distribution of ecosystem effect-sizes was similar between species and between populations (Figure 2), but the difference between species and populations varied considerably among ecosystem metrics (Table 2).

### The multifarious nature of species' ecosystem-effects

We found that copepods differentially affected a broad range of ecosystem characteristics (Figure 2, Table 2). This result is consistent with previous common gardening experiments that used fish [13], [20], [43], and supports the idea that multiple ecosystem metrics are necessary to characterize the diversity of organisms' ecosystem-effects (Figure 2). Just as limiting the number of functional response traits may overestimate the degree of niche conservatism [44], we argue that limiting the number of ecosystem-response metrics can overestimate the similarity of species' ecosystem-effects and the functional redundancy of closely related species and populations. A similar justification for a multivariate description of ecosystems has also been made by researchers studying the relationship between biodiversity and ecosystem functioning [45]. Measuring too few ecosystem-response metrics can underestimate the importance of biodiversity, because species can perform different functions and there can be multifunctional complementarity among species [46], [47]. Our results indicate that different aspects of ecosystem structure (e.g. algae biomass, ammonia concentration) and function (e.g. productivity, sedimentation rate) respond independently to the presence of different species and populations.

One of the most surprising results from our study was that lab-reared and wild-caught populations of one copepod species (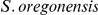) had contrasting effects on multiple ecosystem properties, including ammonia concentrations, gross primary productivity, and sedimentation rate (Figure 4, Table 2). This specific contrast was not part of our original design, and was a byproduct of our inability to culture all three species. The effect of rearing environment is difficult to explain given our current knowledge about the ecology of these species and how they interact with components of aquatic ecosystems. One possibility is that maternal or plasticity effects played a role in generating the observed ecosystem differences. The individuals we used in our experiment were only reared for one generation in the laboratory and were raised on a different food source than they would experience in the wild. Another possibility is that contrasting selective regimes in the wild and the laboratory changed the phenotype distribution of copepods and contributed to the different ecosystem conditions in the experiment. Regardless of the mechanism, we recommend that future studies consider how the rearing and selective environment of organisms used in common gardening experiments might affect ecosystem responses.

Our results suggest that subtle phenotypic differences among species and populations, arising either from natural variation across the landscape or from different rearing environments, can cause significant divergence in the characteristics of ecosystems (Table 2). This conclusion is admittedly tentative because we have not explicitly quantified the phenotypic differences among the copepod species and populations that we used. Copepod taxonomists have described these specific species as being very morphologically similar [26], and previous studies have argued that morphological stasis among copepod species is common, even in clades with deep genetic differences [24]. Nevertheless, phenotypic variation along any number of trait axes (e.g. shape variation, feeding morphology, and physiology) might underly functional differences among species and contribute to the differences in ecosystem-effects that we observed in our experiment.

There is a growing interest in understanding the ecological and ecosystem consequences of phenotypic variation among organisms [13], [18], [20], [48], [49]. If subtle changes in phenotype translate into different ecosystem characteristics, as we observed here (Table 2), then phenotypic evolution could drive variation in the environmental heterogeneity of natural ecosystems. Niche construction theory predicts that variation in species' ecosystem-effects can generate environmental heterogeneity that could promote biological diversity [12], and, ultimately, influence the likelihood of species diversification [14]. However, there is little empirical research aimed at characterizing how organisms affect the scale of environmental variability in ecosystems [50], particularly in a multivariate sense (Table 2). One recent study found that the presence of fish predators can affect the site-to-site variability in species composition of their prey [51], implying that consumers can alter the spatial patterns of biodiversity within ecosystems. Our results suggest that the phenotypic diversity of organisms (both within and among species) can generate heterogeneity in the structure of both the biotic and abiotic environment (Figure 4, Table 2). It is still an open question how such ecosystem-effects shape the selection regimes of other organisms at local and regional scales.

### The size of species' ecosystem-effect contrasts

In our experiment, we found that different copepod groups had different effects on their ecosystems (Table 2; Figure 2), but how do the sizes of these effects compare to other common gardening experiments that used more morphologically divergent organisms? In one such experiment with sticklebacks [13], the size of ecosystem-effect contrasts between species and populations ranged from 0.21–1.33 (mean = 0.53, SD = 0.35, N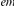 = 9, Table 3) across nine ecosystem metrics (Table 3). For this comparison, we used contrasts between benthic and limnetic stickleback species that exhibit large phenotypic divergence in body shape and feeding morphology [52], and between each species and a population of stickleback with an intermediate phenotype [53]. In comparing the same suite of ecosystem metrics between experiments (Table 2, 3), we found no evidence for a significant difference in ecosystem-effect contrasts between copepod and stickleback species (paired t-test for N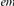 = 9: t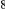 = −0.05, 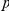 = 0.96). In another comparable experiment, the local adaptation of guppies to contrasting levels of predation by killifish (*Rivulus hartii*), led to divergent effects on stream ecosystems, with contrasts between population treatments ranging from 0.3 to 1.5 (mean = 0.88, SD = 0.57, N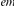 = 5; see Figure 3 in [43]). Again, we found no significant difference between the contrasting ecosystem-effects of different copepod populations and of different guppy populations locally adapted to different predation regimes (two sample t-test: t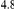 = −1.3, 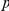 = 0.27). These comparisons across a very limited number of studies provide little evidence for a simple relationship between ecosystem divergence and the phenotypic divergence among species and populations; however, we need many more common gardening experiments to provide a more robust test of this relationship.

**Table 3:** Analysis of contrasts from a stickleback common gardening experiment [13].

Contrast	Algae	Periphyton	Temp	Ammonia	DOC	PAR	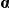 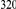	GPP	Decomp	Average
C 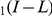	0.66	−0.58	−0.38	−0.66	−0.40	−1.0	−1.82	0.70	−0.26	0.72
C 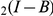	0.79	−0.31	−0.39	−0.02	−0.38	−0.11	−1.62	0.28	−0.50	0.49
C 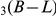	0.06	0.10	−0.05	0.62	0.02	0.92	0.49	−0.18	−0.28	0.30
Avg effect size	0.58	0.31	0.38	0.35	0.21	0.82	1.33	0.36	0.39	

Standardized effect sizes (Cohen's *d*) for ecosystem metrics as in Table 2, calculated for three contrasts between stickleback species. Where 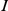 is a population (Cranby Lake) with an intermediate phenotype, 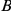 is a benthic species (Paxton Lake) and 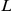 is a limnetic species (Paxton Lake). Averages are based on the absolute values of the contrasts.

There are some important caveats to comparing the ecosystem-effects of organisms across studies. First, there are only a limited number of comparable experiments and so the power of the tests are low. Second, the ecosystem metrics that are used as response variables often differ between studies, making it difficult to determine what aspects of phenotypic divergence (e.g. in foraging morphology) are causing the observed changes to the experimental ecosystems [54]. Third, it is difficult to control for other differences between groups of species that might confound the comparison between the size of phenotypic contrasts and the resulting size of ecosystem-effect contrasts. For example, we did not control for phylogeny in the above comparisons. Copepod species are deeply evolutionary divergent whereas the stickleback and guppy species are more recently diverged. One might predict that more distantly related organisms would have greater ecosystem-effect contrasts than closely related organisms, but this hypothesis remains to be tested.

There are several reasons why the size of ecosystem-effect contrasts is similar between copepod species and between stickleback species (Table 3). One possibility is that the magnitude of phenotypic divergence of functional traits is not strongly correlated with ecosystem divergence. In a previous experiment morphologically divergent species (i.e. limnetics and benthics) had contrasting effects on zooplankton communities in aquatic mesocosms, but the overall ecosystem divergence was greater for contrasts between the intermediate and extreme phenotypes, rather than between the two extreme phenotypes (Table 3). Another possibility is that we may have underestimated the amount of phenotypic divergence among species and populations of freshwater copepods. Although copepod taxonomists regard these species as very morphologically similar [23], [55], these species may differ in several aspects of functional trait variation (e.g. foraging morphology, body shape) that could have ecosystem consequences. In ongoing work we are quantifying how body shape varies among and within Diaptomidae species, in order to better understand the functional significance of morphological variation in copepods (Hausch et al. in progress).

### Evolutionary consequences of variation in species' ecosystem-effects

The phenotypic and genetic diversity of organisms can have a broad range of consequences for ecosystems [54]. Heritable phenotypic variation among individuals can affect species interactions [48], [54] and a wide range of ecosystem processes, such as nutrient cycling [20], rates of decomposition [56], and light transmission [13]. Doing common gardening experiments in combination with a trait-based characterization of phenotypic variation would be a useful way to explore the effects of biological diversity on ecosystem functioning [45], [57], [58] and the evolutionary consequences of phenotypic variation in ecosystems [14].

Phenotypic evolution can affect ecosystems in diverse ways [18], but it is a challenge to understand how the phenotypic traits that underlie community- and ecosystem-effects respond to natural selection [48]. The distinction between traits that affect ecosystems (i.e. ecosystem-effect traits, EETs) and those that underly individual fitness (i.e. functional traits, FTs) is important for understanding the interplay between phenotype evolution and ecosystem dynamics. EETs can include morphological traits (e.g. gape width in fish), life history traits (e.g. growth rate) and stoichiometric traits (e.g. body N:P ratio) [57], and might be the same as, or correlated with, functional traits that underly individual fitness [59]. But, if FTs and EETs are different and uncorrelated, then phenotypic evolution might cause idiosyncratic and cryptic effects on ecosystem dynamics. Although the complexities of interactions between species and their ecosystems (Figure 4, Table 2) can be abstracted using a trait-based approach [58], [60], our results suggest that caution is warranted when equating morphological similarity with functional equivalence in ecosystems.

Quantifying the distribution of ecosystem-effects of organisms across multiple radiations could reveal how the dominant speciation process, namely either ecological speciation or mutation-order speciation [61], influences the structure and dynamics of ecosystems. Non-adaptive radiation, driven by mutation-order speciation [61], produces closely related species that are phenotypically similar and probably functionally equivalent in ecosystems. In radiations where species are primarily differentiated based on secondary sexual traits, we would expect species to have similar resource use requirements and ecosystem impacts, provided that mating preferences are not guided by the environment. Radiations of damselflies [62], salamanders [63], and copepods [24] all exhibit little adaptive phenotypic disparity among species and are good candidates for non-adaptive radiations. Within clades of salamanders, for example, there is little adaptive morphological variation in body size and shape variation among species, and climatic factors limiting geographic ranges are thought to be responsible for diversification via allopatric speciation [64]. Similarly, species of damselflies exhibit marked phenotypic variation in male mating structures [16], but in habitats with fish there is little functional trait variation among co-existing damselfly species [62]. However, resource use and ecosystem impacts have not been thoroughly investigated for these taxa, so it is possible that important features of the interaction between these organisms and their environment have been overlooked.

### Conclusion

Our results suggest that morphological similarity among species and populations may not be a good indication of functional redundancy within ecosystems. As discussed above, this conclusion needs to be tested further with additional phenotypic characterization of the copepod species and populations that we used, and by more investigation of the mechanisms underlying the observed ecosystem-effects. In general, more work is needed to determine whether ecosystem-effect traits are under divergent natural or sexual selection, and whether there are reciprocal interactions between phenotypic evolution and ecosystem change over the course of species radiations. Currently, the ecosystem consequences of phenotypic evolution and species diversification are much less studied than the underlying causes, and so the interplay between species adaption, species diversification, and ecosystem dynamics remains poorly understood.
